# Voluntary stopping of eating and drinking at the end of life – a ‘systematic search and review’ giving insight into an option of hastening death in capacitated adults at the end of life

**DOI:** 10.1186/1472-684X-13-1

**Published:** 2014-01-08

**Authors:** Nataša Ivanović, Daniel Büche, André Fringer

**Affiliations:** 1 Institute of Applied Nursing Sciences St. Gallen, Rosenbergstrasse 59, 9001 St. Gallen, Switzerland; 2 Cantonal Hospital St. Gallen, Palliative Care Center St. Gallen House 33, Rorschacherstrasse 95, 9001 St. Gallen, Switzerland

**Keywords:** Systematic search and review, Voluntary stopping of eating and drinking (VSED), Hastened death, Unbearable suffering, End-of-life, Terminal illness, Terminal care, Palliative care

## Abstract

**Background:**

The terminally ill person’s autonomy and control are important in preserving the quality of life in situations of unbearable suffering. Voluntary stopping of eating and drinking (VSED) at the end of life has been discussed over the past 20 years as one possibility of hastening death. This article presents a ‘systematic search and review’ of published literature concerned with VSED as an option of hastening death at the end of life by adults with decision-making capacity.

**Methods:**

Electronic databases PubMed, EBSCOhost CINAHL and Ovid PsycINFO were systematically searched. Additionally, Google Scholar was searched and reference lists of included articles were checked. Data of the included studies were extracted, evaluated and summarized in narrative form.

**Results:**

Overall, out of 29 eligible articles 16 were included in this review. VSED can be defined as an action by a competent, capacitated person, who voluntarily and deliberately chooses to stop eating and drinking with the primary intention of hastening death because of the persistence of unacceptable suffering. An estimated number of deaths by VSED was only provided by one study from the Netherlands, which revealed a prevalence of 2.1% of deaths/year (on average 2800 deaths/year). Main reasons for patients hastening death by VSED are: readiness to die, life perceived as being pointless, poor quality of life, a desire to die at home, and the wish to control the circumstances of death. The physiological processes occurring during VSED and the supportive care interventions could not be identified through our search.

**Conclusions:**

The included articles provide marginal insight into VSED for hastening death. Research is needed in the field of theory-building and should be based on qualitative studies from different perspectives (patient, family members, and healthcare workers) about physiological processes during VSED, and about the prevalence and magnitude of VSED. Based on these findings supportive care interventions for patients and family members and recommendations for healthcare staff should be developed and tested.

## Background

Preserving autonomy and control at the end of life can be named as one of the last cornerstones of quality of life in situations of unbearable suffering, despite high quality palliative care [1-5]. Between 1958 and 1967 Cicely Saunders achieved improved care and support for the dying patient [6]. The development in the field of hospice and palliative care has significantly improved the situation of people at the end of life [7-10]. A “good death” at home without pain after a long and fulfilled life, with the dying person being at peace with the environment and having at least some control over the events remains a desire for most people [4,11,12]. However, the final weeks or days in human life still remain the greatest challenge for all persons involved (family members, informal caregivers and professional staff) [13]. Unbearable suffering, despite palliative and therapeutic possibilities, leads to requests for ending a patient`s life prematurely. Many aspects of suffering have little in common with symptom control or the use of advanced medicine [14]. Spiritual pain, symptom clusters, bleeding and open wounds, change of body image, social exclusion, and loss of the sense of life are forms of suffering beyond symptom control [14,15].

Hence, over the past 20 years voluntary stopping of eating and drinking (VSED) at the end of life has been discussed as one possibility among several to preserve autonomy, to retain control, and to hasten death without infringing the fundamental ethical principles of Western society [14,16,17]. But, the wish to end one’s life prematurely seems to be incomprehensible for people who have never been confronted with unbearable suffering in their family or social environment. Moral conflicts of clinicians and nurses lead to the fact that VSED remains a marginal topic in the field of palliative care [18-20]. In addition, there is the assumption among some healthcare professionals that VSED leads to more suffering and additional strain for the patient [21]. Furthermore, food intake is associated with a high social value and is considered as a symbol of social participation. Therefore, stopping eating and drinking can be misunderstood by family members as denial [21]. As Schwarz pointed out: [22]*“The desire for a hastened death regularly occurs, but such thoughts are frequently kept secret by patients unless clinicians specifically inquire”.* But it is not uncommon that terminally ill cancer patients ask their caregivers for assisted or hastened death [23-25]. Within the scientific community and in research the possibility of a hastened death is often not taken into account [8]. These aspects may explain why VSED has hardly been examined in the past 20 years, but still remains a legal option in some Western societies [17,26].

Our understanding of palliative care is based on the definition from the World Health Organization (WHO) [27,28]. Against this background, to fully understand what it means to die remains concealed unless we are affected by death [29]. Professionals have to develop attitudes that will be of help, in a holistic manner, for the persons concerned [30]. Therefore, not only is symptom control of central importance, but so is protecting the patient’s autonomy and the ability to maintain control as final aspects of quality of life. In the context of professional palliative care, VSED is highly relevant and therefore requires further analysis.

The international perspective shows that options to hasten death have been a political topic only in some countries where VSED is regarded as a legal possibility to hasten death. For example, comprehensive discussions where lead in Oregon (USA) and the Netherlands [17,31,32]. Switzerland can be called a “right-to-die” society [32]. Commercial organizations such as DIGNITAS (established in 1998) and EXIT (established in 1982) offer assisted suicide to die with dignity. In 2013 personal rights in Switzerland were strengthened by a further amendment. In this context it can be assumed that the topic of assisted suicide will gain relevance in society and healthcare. Despite the long debate about suicide, it is surprising that VSED so far has not been a topic of debate in Swiss society.

Against this background, in 2012 a mapping review was conducted to explore the phenomenon of VSED [33]. Knowledge about ways to end one’s life prematurely and clarifying the role of VSED helps caregivers respond to patients’ requests in a professional manner. In the context of the “End of Life National Research Programme NRP 67” in Switzerland, the exploration and explanation of VSED is essential as demand is made for increased knowledge in the areas of “dying processes and provision of care”, “decisions, motives and attitudes”, “regulations and proposals for action”, and “cultural concepts and social ideals” [34]. Analysis of the current scientific knowledge reveals gaps in the existing research. Therefore, it is necessary to conduct a ‘systematic search and review’ in order to use the current scientific knowledge as a basis for further empirical work. The present article is the first ‘systematic search and review’ about VSED.

## Methods

A systematic search and review [35] was performed to give a comprehensive overview about VSED as an option to hasten death in adults with decision-making capacity at the end of life. This includes (1) clarifying the definition, the prevalence and magnitude, and the ethical aspects and moral standards of VSED, (2) exploring the experience of patients, family members and healthcare professionals with VSED, (3) explaining the physiological processes during VSED and (4) identifying accurate interventions for healthcare professionals to support patients during the process of VSED.

We conducted a systematic literature search for English and non-English articles according to the PRISMA guideline [36] in the following databases: PubMed (1947–2013), EBSCOhost CINAHL (1981–2013) and Ovid PsycINFO (1967–2013). Database searches were completed between October 2012 and March 2013. Prior to that, a pre-search for sensitizing relevant key words was performed independently by two assistants. The PubMed search was developed by one author (NI), checked by a second author (AF), and translated for use in other databases. The PubMed search string can be viewed in Additional file 1. All database search strings contained both controlled vocabulary and free text words representing the concept of VSED at the end of life. An additional search was performed in the internet using Google Scholar. Furthermore, we checked all reference lists of the included articles for additional published research.

Research articles were included if they report on VSED and adults with decision-making capacity at the end of life, as well as family members and healthcare professionals who have experience with VSED. Furthermore, articles met the inclusion criteria if they described the physiological process during VSED and supporting interventions by healthcare professionals. As VSED is an ethically controversial issue, discussion contribution papers were also included. Articles were excluded if they focused on VSED indirectly, referred to patients in vegetative states or patients with an inability to eat and drink because of disease, patients with artificial nutrition, and when VSED was politically motivated (e.g. hunger strikes). Newspaper articles and commentary letters to the editor of journals were also excluded, because they were regarded as information not contributing to our research questions.

Two authors (NI and AF) independently screened the titles and abstracts for eligibility. Full reports were obtained if the abstracts met our inclusion criteria or when no abstract was available. The full-text of relevant articles were read independently by two authors (NI and AF) to check for inclusion. Disagreements were resolved by discussion. Data were primarily extracted by one author (NI) and checked by the second author (AF) using a data extraction sheet classified with respect to design, objective, sample, measures, analysis and results/case description.

Because the included articles used a wide variety of descriptive and quantitative methods, we evaluated them according to general criteria for quantitative research as described by Coughlan et al. [37]. There was no uniform rating scale and the articles were coded according to two criteria proposed by Whittemore [38] when using diverse empirical sources: methodological rigour and data relevance on a 2-point scale (1 = low; 2 = high). Based on the data, evaluation articles with low rigour or relevance were not excluded, but rather considered as trends.

Data synthesis was performed in narrative form typically used for ‘a systematic search and review design’ [35].

## Results

**Figure 1:**
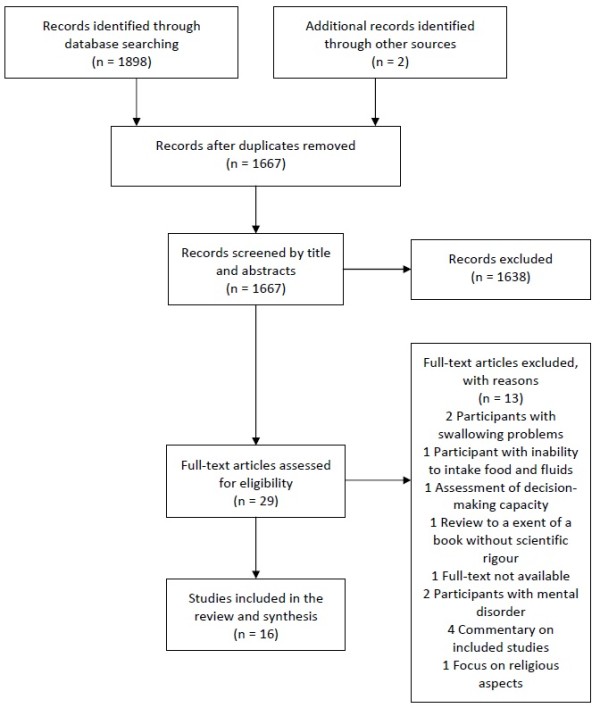
**Flow chart.** Flow chart on study selection process according to the PRISMA Guideline [36].

We identified 29 relevant articles. Of these, 16 articles were included in this review (4 survey studies, 4 case reports and 8 narrative reviews). The selection process with reasons of the excluded studies can be viewed in Figure 1. Due to heterogeneity of the study designs and mostly weak methodological quality, the results have to be interpreted with caution and refer more to tendencies. The Additional file 2: Table S1 gives an overview of the included articles and the evaluation.

### Definition and magnitude of voluntary stopping of eating and drinking

Three survey studies [17,19,31], four case reports [14,22,39,40] and six reviews [16,18,20,41-43] stated a description of the concept of VSED. In the included articles different terms are used to describe the concept of VSED, but they all have the same meaning and clearly express criteria that distinguish VSED from other forms of forgoing life-sustaining measures in palliative care (e.g. medical treatment) and assisted suicide. VSED is described as an action of a competent, capacitated person, who voluntarily and deliberately chooses to stop eating and drinking with the primary intention to hasten death because unacceptable suffering persists [14,20,31,41]. Therefore, VSED is related to patients without cognitive impairments [19]. From this perspective, the patient is physically able to orally take in food and fluids, but consciously is not willing to do so [41]. Under these circumstances VSED differs from other reasons for stopping eating and drinking such as loss of appetite, inability to eat and drink or disinterest in food and drinks, which are often present at the end of life, [31,39] because discipline and stamina are part of the decision and execution of VSED [14,18,19]. The conscious wish for VSED is an expression of control by highly competent patients and cannot simply arise from a situational whim [18]. VSED could be an option to hasten death in situations where palliative measures are ineffective. For this reason, VSED focuses on patient groups with irreversible terminal illnesses [14,16,18]. VSED can thus be considered as a waiver of life-sustaining measures and it is a legal method like pain management or forgoing life-sustaining therapy [20]. VSED is also an alternative to physician-assisted suicide (PAS) and voluntary active euthanasia (VAE) [14]. In connection with VSED, several synonyms are used such as voluntary refusal of food and fluids (VRFF) or patients’ refusal of hydration and nutrition (PRHN) [16,17]. In the following we use the term VSED, as described above.

The scope of VSED was only investigated by Chabot & Goedhart [17] in a population-based prevalence study in the Netherlands. They asked relatives, friends or nurses from deceased patients (N = 31,516) whether they were a confident in the decision of the patients to hasten death by VSED. Of these, 839 people indicated that they were confident, but only 97 deaths by VSED were confirmed. The estimated number of deaths by VSED over the period from 1999 to 2003 was on average 2.1% (range 1.4%–2.09%) or 2800 (range 1700–3900) deaths/year. These authors assumed that the number of deaths by VSED confirmed the hypothesis that the wish to control the time of one’s death is steadily increasing [17].

### Ethical aspects and moral standards

In the literature regarding VSED some strengths and advantages are described. The included case reports draw a largely positive picture of VSED ─ not only because it helps to stop unimaginable suffering, but also because it is an expression of control and autonomy [22,40]. The aspect of time offers that the person concerned may always reconsider the decision and it allows for the social environment to mentally prepare for this situation [16,39]. Moreover, the process of VSED contains the possibility to resolve communication errors or misunderstandings in the decision-making process between patient and professionals [16]. Additionally, from an ethical point of view VSED has the advantage of being neither physician-ordered nor -directed [14]. According to this, authors such as Jansen [18] demand: “If a competent patient’s decision to engage in VSED is voluntary, his or her physician is morally and legally required to respect it”. Since VSED causes no bureaucratic barriers, it seems that hastening death by VSED is faster compared to PAS or VAE [16]. A disadvantage of VSED is the fact that persons involved in the patient’s decision-making process often perceive VSED as repulsive, because they consider it unethical. Furthermore, there is the danger that the decision for VSED underlies a subtle constraint [20]. The long period between starting VSED and death (sometimes several weeks) may turn out to be a disadvantage [20,40,43].

### Patients’, family members’ and healthcare professionals’ perspective on VSED

To date no qualitative study has comprehensively investigated patients’ experiences with VSED at the end of life. Four case reports included in this review tried to explore patients’ perspectives on VSED [22,39,40,42]. These reports described how patients express their wish to die and how the decision for VSED was taken. Reasons leading to the request for hastening death were investigated. In most cases, due to a deterioration of health status or a progression of the medical condition, the burden of living outweighed any associated benefit for the patient without any perspective or reason for life. Furthermore, it is mentioned that palliation or symptom management is ineffective and cannot relieve discomfort, which reinforces the wish to hasten death. Some patients expressed it as “being tired of life” or “having it done”. Under these circumstances patients reported that they viewed themselves as a burden to their family members, which also influences their decision for hastened death. The decisive factors for choosing VSED instead of other methods is justified by the fact that patients have self-control over their deaths and act in a self-determined way. VSED enables patients to decide for themselves at which time the process should start. Moreover, as they are fully informed about VSED, they know that it is legal and their family members will not be confronted with any juridical consequences related to their decision.

Similar results have been shown in a survey study by Ganzini et al. [31], who examined the perception of hospice nurses who have experience with VSED. The survey was conducted by means of mailed questionnaires. The study included 429 hospice nurses from 52 certified home hospice programmes in Oregon, USA. Of those, 126 (41%) nurses reported on the most common reasons for hastening death, which were: readiness to die, the belief that continuing to live was pointless, poor quality of life, a desire to die at home, and the wish to control the circumstances of death.

Another survey study by Harvath et al. [19] reported on hospice workers’ attitudes towards VSED. This study was also conducted in Oregon, USA. Of the 545 eligible hospice workers, 390 (307 nurses and 83 social workers) responded to the questions in a mailed questionnaire. The survey questions addressed the support for VSED and different statements about VSED. If a patient chose to hasten death by VSED, 95.4% of the hospice workers indicated that they would still continue to care for the patient. More than three-quarters of the respondents were of the opinion that VSED should be an option if physical, psychological or spiritual suffering exists. The respondents indicated that if they themselves were to become terminally ill, 70.7% would even consider choosing VSED as an option for themselves.

A study by Mattiasson et al. [44] included 189 nursing home staff from 13 nursing homes in Sweden, and explored nurses’ understanding of problem solving with regard to residents without cognitive impairment who refuse to eat and drink. A self-report questionnaire with two main questions was completed by 157 nurses. Of the 157 nurses, 92 considered that the patient’s autonomy should be respected, and “if a person does not want to live, he/she should decide – even if this can cause confusion” [44].

### Physiological processes during VSED

No study was found that explicitly examined physiological processes during VSED in adults at the end of life. In the existing literature it is mentioned that the dying process will often take several days or weeks, depending on the patient’s condition [20,40-42]. Chabot & Goedhart [17] assumed that if death occurred less than 7 days after starting with VSED, then death could be attributed to the underlying disease. Furthermore, death by VSED is described as a peaceful and comfortable death, and terminally ill patients dying of dehydration or starvation do not suffer if adequate palliative care is provided [16,31,40]. There is the assumption that the accompanying symptom “thirst” triggered by VSED is rather a sensation of dryness than the need to ingest fluids, which can be relieved by oral care [21].

### Interventions by healthcare professionals to support patients during VSED

The literature search yielded no experimental or quasi-experimental study that examined interventions to support patients during VSED at the end of life. The review by Quill et al. [14] provides a potential clinical guideline, which describes elementary cornerstones for healthcare professionals to support patients during VSED. The cornerstones are based on safeguards typically proposed for regulating VAE and PAS in terminally ill patients [20]. From the authors’ perspective VSED should be provided as a last-resort option, if available excellent palliative care is unable to relieve current suffering. One main cornerstone of the guideline is to obtain the patient’s informed consent, including the assessment of the patient’s capacity that he or she comprehends the treatment and possible alternatives. This includes also obtaining a second opinion of an expert in palliative care, mental health, or of a specialist in the patient’s underlying disease in order to exclude depression or other mental disorders. Furthermore, the authors recommend involving both family members and healthcare providers in the decision making process, as moral conflicts may occur. To provide adequate palliative care for the patient during the process of VSED, staff consent is needed. If providers perceive a moral conflict for themselves, they should refer the patient to other professionals. Finally, to ensure accountability, processes of documentation and reporting should be specified. Similar demands were made by other authors [16,39-41,45]. It is recommended that VSED should be carried out mainly in the context of professional palliative care monitoring and support, as stopping eating and drinking may lead to new symptoms that require clarification and palliation [14]. Supportive interventions during the VSED process such as terminal sedation (TS) are controversially discussed, because the benefits remain unclear [14,43].

## Discussion

The aim of this systematic search and review was to gain a comprehensive insight into voluntary stopping of eating and drinking as an option to hasten death in adults with decision-making capacity at the end of life. The intensive examination of the literature shows that the subject under study has been marginally researched and that there is no scientific basis on which VSED could be explained in all of its dimensions. Therefore, recommendations for its practice in the palliative care setting could not be drawn.

One aim of the present study was to clarify the definition and magnitude of VSED. Based on the available literature, a definition could be sketched, but important basic research to precisely depict the subtleties of VSED as a concept in a comprehensive manner is missing. The available articles can indeed be described as heterogeneous and inconclusive. They represent a patchwork rather than a picture. VSED can be defined as an action by a competent, capacitated person, who voluntarily and deliberately chooses to stop eating and drinking with the primary intention to hasten death because unacceptable suffering persists [14,20,31,41]. But what does it mean if a “capacitated person” at the end of life is experiencing unbearable suffering? The definition of VSED described above seems to be clear, but the difference as compared to other forms of voluntary renunciation of food and fluid intake requires further investigation. Explaining the magnitude of VSED poses a problem as this concept is mainly discussed in the available literature [17,19,20,43] in the context of active and passive euthanasia. In our analysis it became clear that in the debate on VSED a fine line exists concerning the ethical conflict between respect for patients and beneficence.

In exploring how patients, family members and healthcare professionals experience VSED, it becomes obvious that the most positive aspect of VSED consists in the preservation of the patients’ autonomy and control regarding their own life. As studies in the palliative setting show, the aspects of autonomy and self-control play an increasingly important role [4,45]. In addition, there is little evidence that VSED is considered as an alternative way to hasten death among healthcare professionals in palliative and hospice care in Oregon [19]. Specific conclusions about the significance of VSED from the perspective of patients, family members and healthcare professionals cannot be made at this point as qualitative studies are still missing. However, it is clear in the debate that physicians are not absolutely necessary to perform VSED, but the situation of the target patients for VSED with unbearable suffering necessarily requires palliative care treatment. This is a contradiction in terms, because on the one hand VSED can be performed independently by patients, but on the other hand the persons concerned need intense and excellent medical and palliative care support [14,31]. The active discussion on VSED varied depending on the cultural and regional context. Since VSED is regulated legally in Oregon and the Netherlands, this has mainly been considered in these regions. Thus, this leads inevitably to a cross-regional bias. The authors of this study concluded that the decision for hastened death has to be respected if it is not influenced by mental health problems. For this reason VSED can be interpreted as a patient’s decision against life-sustaining measures.

Objective measures of VSED were not possible to assess, because studies on physiological processes during VSED are missing.

Interventions for healthcare professionals to support patients during the process of VSED could not be identified. Contrary to the recommendations in the included articles, we think that the issue of suicide, euthanasia and hastened death should not be regarded as a last-resort option. They have to be discussed early with the affected persons and not in the last days of life. If options of prematurely ending one’s life are known beforehand, VSED is an expression of autonomy and control, and therefore a sign of the patient’s competence.

### Limitations

The benefit of this work consists in the fact that it offers the first ‘systematic search and review’ on the topic of VSED. Compared to other reviews, this article provides the basis for further empirical research. The limitation of this review lays less in the methodological execution, but rather the availability of relevant literature. As shown in the results, VSED is nearly unexplored. Since the synthesis of results is mainly based on narrative reviews, case reports and a few survey studies, the results must be treated with caution and potential risk of bias about the topic should be taken into account. From the literature it became clear that the ethical and legal aspects of VSED require a more detailed analysis in the dependence of the respective settings.

## Conclusions

The existing evidence concerning VSED at the end-of-life is intertwined by the authors of the included articles to such a degree that a clear and independent appraisal of the available literature cannot be guaranteed. The evidence can be described as continuous interweaving of published articles. In this respect, we conclude that the evidence was artificially reproduced over time through repeated citations of narrative reviews without new insights based on original studies. The demand expressed by Bernat et al. [16] with regard to phenomenological studies, that is qualitative studies on the subject and a systematic examination of physiological processes during VSED, has hardly been fulfilled. Recommended steps for further research on VSED are:

(1) Extended qualitative research is needed for theory-building, especially studies based on Grounded Theory. Furthermore, observational studies and qualitative expert interviews based on qualitative theories, as well as representative epidemiological studies, are necessary to determine the extent and distribution of VSED.

(2) Based on the findings supportive care interventions for patients and family members need to be developed.

(3) The development of clinical guidelines is required and recommendations should consider cultural and social requirements.

In summary, it can be stated that the importance of VSED for patients is obvious, because they get an additional option to hasten death. VSED reflects all 12 principles of a ‘good death’, which was defined by the Debate of the Age Health and Care Study Group and involves, for example, to be able to retain control of what happens, to be able to leave when it is time to go, or the possibility to say good bye [4].

## Abbreviations

VSED: Voluntary stopping of eating and drinking; VRFF: Voluntary refusal of food and fluids; PRHN: Patients’ refusal of hydration and nutrition; PAS: Physician-assisted suicide; VAE: Voluntary active euthanasia; TS: Terminal sedation.

## Competing interest

The authors declare that they have no competing interests. This research was funded by the Swiss Academy of Medical Sciences and the Käthe Zingg Schwichtenberg Foundation.

## Authors’ contributions

The conception and design of the study were made by NI and AF. The manuscript was drafted by NI and AF and critically revised by DB. The final version of the manuscript for submission was approved by NI, DB, and AF.

## Pre-publication history

The pre-publication history for this paper can be accessed here:

http://www.biomedcentral.com/1472-684X/13/1/prepub

## Supplementary Material

Additional file 1 **Search strategy.** PubMed. Click here for file

Additional file 2: Table S1 Overview about the included articles and the evaluation. Click here for file

## Statement of adherence to the PRISMA guidelines

The authors of this article state that they have adhered to the PRISMA guidelines (2009) for systematic reviews and meta-analyses.

## References

[B1] Winzelberg GS Hanson LC Tulsky JA Beyond autonomy: diversifying end-of-life decision-making approaches to serve patients and families J Am Geriatr Soc 2005 53 6 1046 1050 10.1111/j.1532-5415.2005.53317.x 15935032

[B2] Chochinov HM Dying, dignity, and new horizons in palliative end-of-life care CA Cancer J Clin 2006 56 2 84 103 10.3322/canjclin.56.2.84 16514136

[B3] Patrick DL Curtis JR Engelberg RA Nielsen E McCown E Measuring and improving the quality of dying and death Ann Intern Med 2003 139 5 Pt 2 410 415 12965967 10.7326/0003-4819-139-5_part_2-200309021-00006

[B4] Smith R A good death. An important aim for health services and for us all BMJ 2000 320 7228 129 130 10.1136/bmj.320.7228.129 10634711 PMC1128725

[B5] Dees M Vernooij-Dassen M Dekkers W van Weel C Unbearable suffering of patients with a request for euthanasia or physician-assisted suicide: an integrative review Psychooncology 2010 19 4 339 352 10.1002/pon.1612 19771571

[B6] Clark D ‘Total pain’, disciplinary power and the body in the work of Cicely Saunders, 1958–1967 Soc Sci Med 1999 49 6 727 736 10.1016/S0277-9536(99)00098-2 10459885

[B7] Saunders C The evolution of palliative care Patient Educ Couns 2000 41 1 7 13 10.1016/S0738-3991(00)00110-5 10900362

[B8] Meier DE Meier DE, Isaacs SL, Hughes RG The development, status, and future of palliative care Palliative Care. Transforming The Care of Serious Illness 2010 San Francisco: Jossey-Bass 3 76

[B9] Lentz J Sherman D Matzo M, Sherman D Development of the specialty of hospice and palliative care nursing Palliative Care Nursing. Quality Care at the End of Life 2010 New York: Springer 107 117

[B10] de Conno F Blumhuber H Rocafort J Bruera E, Higginson IJ, Ripamonti C, von Gunten C The development of palliative medicine in Europe Textbook of palliative medicine 2009 London: Hodder Arnold 12 21

[B11] Seale C van der Geest S Good and bad death: introduction Soc Sci Med 2004 58 5 883 885 10.1016/j.socscimed.2003.10.034 14732602

[B12] Gomes B Higginson IJ Where people die (1974–2030): past trends, future projections and implications for care Palliat Med 2008 22 1 33 41 10.1177/0269216307084606 18216075

[B13] Lokker M Zuylen L Veerbeek L Rijt CD Heide A Awareness of dying: it needs words Support Care Cancer 2012 20 6 1227 1233 10.1007/s00520-011-1208-7 21688164 PMC3342506

[B14] Quill TE Byock IR Responding to intractable terminal suffering: the role of terminal sedation and voluntary refusal of food and fluids. ACP-ASIM End-of-Life Care Consensus Panel. American College of Physicians-American Society of Internal Medicine Ann Intern Med 2000 132 5 408 414 10.7326/0003-4819-132-5-200003070-00012 10691593

[B15] Aktas A Walsh D Rybicki L Symptom clusters and prognosis in advanced cancer Support Care Cancer 2012 20 11 2837 2843 10.1007/s00520-012-1408-9 22361827

[B16] Bernat JL Gert B Mogielnicki RP Patient refusal of hydration and nutrition. An alternative to physician-assisted suicide or voluntary active euthanasia Arch Intern Med 1993 153 24 2723 2728 10.1001/archinte.1993.00410240021003 8257247

[B17] Chabot BE Goedhart A A survey of self-directed dying attended by proxies in the Dutch population Soc Sci Med 2009 68 10 1745 1751 10.1016/j.socscimed.2009.03.005 19375206

[B18] Jansen LA No safe harbor: the principle of complicity and the practice of voluntary stopping of eating and drinking J Med Philos 2004 29 1 61 74 10.1076/jmep.29.1.61.30413 15449813

[B19] Harvath TA Miller LL Goy E Jackson A Delorit M Ganzini L Voluntary refusal of food and fluids: attitudes of Oregon hospice nurses and social workers Int J Palliat Nurs 2004 10 5 236 241 15215708 10.12968/ijpn.2004.10.5.13072

[B20] Quill TE Lo B Brock DW Palliative options of last resort: a comparison of voluntarily stopping eating and drinking, terminal sedation, physician-assisted suicide, and voluntary active euthanasia JAMA 1997 278 23 2099 2104 10.1001/jama.1997.03550230075041 9403426

[B21] Byock I Patient refusal of nutrition and hydration: walking the ever-finer line Am J Hosp Palliat Care 1995 12 2 8 9–13 10.1177/104990919501200205 7605733

[B22] Schwarz JK Death by voluntary dehydration: suicide or the right to refuse a life-prolonging measure? Widener Law Rev 2011 17 351 361

[B23] Matzo ML Schwarz JK In their own words: oncology nurses respond to patient requests for assisted suicide and euthanasia Appl Nurs Res 2001 14 2 64 71 10.1053/apnr.2001.22371 11319701

[B24] Block SD Billings JA Patient requests to hasten death. Evaluation and management in terminal care Arch Intern Med 1994 154 18 2039 2047 10.1001/archinte.1994.00420180041005 7522432

[B25] Volker DL Oncology nurses’ experiences with requests for assisted dying from terminally ill patients with cancer Oncol Nurs Forum 2001 28 1 39 49 11198896

[B26] Eddy DM A piece of my mind. A conversation with my mother JAMA 1994 272 3 179 181 10.1001/jama.1994.03520030013005 8022025

[B27] Sepúlveda C Marlin A Yoshida T Ullrich A Palliative care: the World Health Organization’s global perspective J Pain Symptom Manage 2002 24 2 91 96 10.1016/S0885-3924(02)00440-2 12231124

[B28] van Kleffens T van Baarsen B Hoekman K van Leeuwen E Clarifying the term ‘palliative’ in clinical oncology Eur J Cancer Care 2004 13 3 263 271 10.1111/j.1365-2354.2004.00481.x 15196230

[B29] Neimeyer RA Wittkowski J Moser RP Psychological research on death attitudes: an overview and evaluation Death Stud 2004 28 4 309 340 10.1080/07481180490432324 15129688

[B30] Youll JW The bridge beyond: strengthening nursing practice in attitudes towards death, dying, and the terminally ill, and helping the spouses of critically ill patients Intensive Care Nurs 1989 5 2 88 94 10.1016/0266-612X(89)90030-8 2754230

[B31] Ganzini L Goy ER Miller LL Harvath TA Jackson A Delorit MA Nurses’ experiences with hospice patients who refuse food and fluids to hasten death N Engl J Med 2003 349 4 359 365 10.1056/NEJMsa035086 12878744

[B32] Frei A Schenker TA Finzen A Kräuchi K Dittmann V Hoffmann-Richter U Assisted suicide as conducted by a “Right-to-Die”-society in Switzerland: a descriptive analysis of 43 consecutive cases Swiss Med Wkly 2001 131 25–26 375 380 11524903 10.4414/smw.2001.09725

[B33] Klein U Fringer A Freiwilliger Verzicht auf Nahrung und Flüssigkeit in der Palliative Care: ein Mapping Review Pflege Zin press 10.1024/1012-5302/a000329 24297830

[B34] Zimmermann-Acklin M Deliens L Felder S Käppeli S Pautex S Perrier A Streckeisen U Tag B Lebensende, Nationales Forschungsprogramm NFP 67, Ausführungsplan 2011 Bern: Schweizerischer Nationalfond

[B35] Grant MJ Booth A A typology of reviews: an analysis of 14 review types and associated methodologies Health Info Libr J 2009 26 2 91 108 10.1111/j.1471-1842.2009.00848.x 19490148

[B36] Moher D Liberati A Tetzlaff J Altman DG Preferred reporting items for systematic reviews and meta-analyses: the PRISMA statement BMJ 2009 339 b2535 10.1136/bmj.b2535 19622551 PMC2714657

[B37] Coughlan M Cronin P Ryan F Step-by-step guide to critiquing research. Part 1: quantitative research Br J Nurs 2007 16 11 658 663 17577184 10.12968/bjon.2007.16.11.23681

[B38] Whittemore R Knafl K The integrative review: updated methodology J Adv Nurs 2005 52 5 546 553 10.1111/j.1365-2648.2005.03621.x 16268861

[B39] Schwarz JK Stopping eating and drinking Am J Nurs 2009 109 9 52 61 10.1097/01.NAJ.0000360314.69620.43 19704237

[B40] Berry ZS Responding to suffering: providing options and respecting choice J Pain Symptom Manage 2009 38 5 797 800 10.1016/j.jpainsymman.2009.09.001 19896573

[B41] Schwarz J Exploring the option of voluntarily stopping eating and drinking within the context of a suffering patient’s request for a hastened death J Palliat Med 2007 10 6 1288 1297 10.1089/jpm.2007.0027 18095807

[B42] Quill TE Lee BC Nunn S Palliative treatments of last resort: choosing the least harmful alternative. University of Pennsylvania Center for Bioethics Assisted Suicide Consensus Panel Ann Intern Med 2000 132 6 488 493 10.7326/0003-4819-132-6-200003210-00011 10733450

[B43] Rady MY Verheijde JL Distress from voluntary refusal of food and fluids to hasten death: what is the role of continuous deep sedation? J Med Ethics 2012 38 8 510 512 10.1136/medethics-2011-100278 22038559

[B44] Mattiasson AC Andersson L Staff attitude and experience in dealing with rational nursing home patients who refuse to eat and drink J Adv Nurs 1994 20 5 822 827 10.1046/j.1365-2648.1994.20050822.x 7745172

[B45] Yale SL Dying patients who refuse nutrition and hydration: holistic nursing care at the end of life Altern Complement Ther 2005 11 2 100 102 10.1089/act.2005.11.100

