# Supplementary material for: Voluntary stopping of eating and drinking at the end of life – a ‘systematic search and review’ giving insight into an option of hastening death in capacitated adults at the end of life
Source: BMC Palliat Care. 2014 Jan 8;13:1. doi: 10.1186/1472-684X-13-1 (PMC3893440; doi:10.1186/1472-684X-13-1)
Supplement: Additional file 1 — Search strategy. PubMed. [file 1472-684X-13-1-S1.pdf]

**Additional file 1:**

*Search strategy: PubMed*
((((((((("Withholding Treatment"[Mesh])) OR ("Treatment Refusal"[Mesh])) OR ("Right to die"[Mesh])) OR ("Euthanasia, Passive"[Mesh])) OR ("Suicide, assisted"[Mesh])) OR ("Euthanasia, Active, Voluntary"[Mesh])) OR (hasten death)) OR (desire to die))) AND (((((((("Fasting"[Mesh])) OR ("Eating"[Mesh])) OR ("Drinking"[Mesh])) OR ("stopping eating and drinking")) OR ("refusal of food and fluids")) OR (nutrition)) OR (hydration)) OR (food)) AND (Humans[Mesh])
