# Supplementary material for: Voluntary stopping of eating and drinking at the end of life – a ‘systematic search and review’ giving insight into an option of hastening death in capacitated adults at the end of life
Source: BMC Palliat Care. 2014 Jan 8;13:1. doi: 10.1186/1472-684X-13-1 (PMC3893440; doi:10.1186/1472-684X-13-1)
Supplement: Additional file 2: Table S1 — Overview about the included articles and the evaluation. [file 1472-684X-13-1-S2.pdf]

Table 1: Overview about the included articles and the evaluation

Survey studies

Authors, nationality	Objective	Sample	Data collection / Measurement	Analysis	Results	Conclusion	Evaluation Method. Quality	Relevance
Chabot & Goedhart (2009) Netherland [17]	Isolating VRFF[1] and ILMC[2] from other dying trajectories	Adults (relatives, friends or nurses from died patients) n = 31'516<br><br>*Exclusion criteria:*<br>-Communication not possible (patient)<br>-Death after less than 7 days of VRFF[1] occurred	Two-stage screening procedure<br>-*Stage 1:* broad screening based on 4 questions<br>-*Stage 2:* 100 pre-structured questions and five open-ended questions	-Frequency by standard weighting method<br>-Poisson distribution for 95% CI<br>-Alpha-level: .01	839 persons indicated to be a confident in VRFF[1]; by the second screening 97 proxies remained<br>Confidents rated the dying process by VRFF[1] in about 75% cases as "dignified death".<br>Estimated number of deaths by VRFF[1] per Year in Netherland over 1999-2003: 2800 (1700-3900)/year; 2.1% (1.4%-2.09%)/year	The number of deaths by VSED confirmed the hypothesis that the wish to control the time of one's death has grown in importance in the Netherlands.	2	2
Ganzini et al. (2003) Oregon, USA [31]	Describing the experience of hospice nurses with VRFF[1]	Nurses n = 429<br><br>*Setting:* certified home hospice programs	Mailed questionnaire:<br>-21 possible reasons why the patient stopped intake of food and fluids<br>-Patient's overall peacefulness, suffering and quality of the process<br>-Comparing the characteristics of family caregivers	-*Categorical*: frequencies and proportions<br>-*Continuous:* medians and interquartile ranges<br>-Group comparisons with Mann-Whitney-U-test or Student's-t-test<br>-p-Value two-sided; alpha level .05	126 (41%) reported on one patient who chose VRFF[1]<br>*Common reasons for hastening death:*<br>Readiness to die, continuing to live was pointless, quality of life poor, a desire to die at home, desire to control the circumstances of death<br><br>Nurses rated those who died by VRFF[1] as suffering less and being more at peace.<br>85% of patients died within 15 days after VRFF[1]	Patients choosing VRFF[1] usually die a "good" death within two weeks	1	2
Harvath et al. (2004) Oregon, USA [19]	Hospice worker's attitudes towards VRFF[1] and PAS[3]	Nurses and social workers n = 545<br><br>*Setting:* certified home hospice programs	Mailed questionnaire:<br>-Demographic data<br>-3 Items asked about the respondents' support or opposition for VRFF[1] / PAS[3]<br>-Agreement with 10 statements regarding VRFF[1] / PAS[3]	-*Categorical*: frequencies and proportions: group comparisons with x^2-test<br>-*Continuous*: means with standard deviation; group comparison with t-test	307 of nurses (71%) and 83 of 116 social workers (78%) returned the survey.<br><br>Hospice workers expressed support for patients who choose to hasten death by VRFF[1].<br>Only 2.6% indicated that they thought VRFF[1] was immoral or unethical.	Perceptions regarding VRFF[1] are significantly different than those regarding PAS[3].	1	2
Mattiasson & Andersson (1994) Sweden [44]	Understanding of problems regarding nursing home patients	Nursing home personnel (nurses, assistant nurses, nurses' aides, and	Questionnaire with case description:<br>(a) What would your unit's decision be in this case?	Content analysis method by Polit & Hungler (1997)<br>Inter-rater agreement by	Answer to question (a) was that 20% reported that the patient's autonomy would be respected.<br>Answer to question (b) was that almost 50%	Staff with long experience of working in medical care tend to be the	1	2

who are rational and refuse to eat and drink	extra staff) n = 189	(b) What is your personal opinion in this case?	Miles & Hubermann (1984)	have the opinion that the patient's autonomy must be respected.	most supportive of the patient's wishes.

[1]VRFF = Voluntary refusal of food and fluids; [2]ILMC = independently taking Lethal Medication attended by a Confidant; [3]PAS = Physician-assisted suicide

Case reports

Authors, nationality	Sample	Case description	Conclusion	Evaluation Method. Quality	Relevance
Berry (2009) Vermont, USA [40]	A 52-year-old woman with progressive right-sided hemidystonia accompanied by a painful pressure-like pulsation through the right side of her body	She admitted to an inpatient hospice for supportive care and symptom management with the understanding that her choice to stop eating and drinking would be honoured.<br>She died on her 17th day of her fast.<br>After her death, her family expressed profound gratitude to the hospice staff for being respectful of her wishes.	An example that the refusal of food and fluids could be an option for terminally ill people to peacefully hasten death	1	2
Quill et al. (2000) USA [14]	83-year-old woman admitted to a nursing facility one year after experiencing a major cerebrovascular accident that left her with a dense hemiparesis but retained cognition.	She had no moral objection to voluntarily hastening death, as she being a life-long Unitarian.<br>Through reading the newspaper about a case of a woman who had chosen VSED[1], she began to explore this option with her family and physician.<br>Several staff members were unable to accept her choice.<br>She died on her 15th day of her VSED[1] process.	VSED[1] is legal, but it is a more extraordinary option; should be considered only when no acceptable alternatives are available; both patient and physician should consider participation to be moral.	1	2
Schwarz (2009) USA [39]	99-year-old woman; was not terminally ill but the quality of life was significantly diminished by many chronic ailments	She was tired of life and was ready to leave after years of living with these chronic conditions.<br>She asked her hospice nurse for advice how she could end her life. The hospice nurse referred her and her family to a non-profit end-of-life organization.<br>After several meetings and much discussion about end-of-life options, she elected VSED[1].<br>She died peacefully on her 10th day of her fast.	The process of VSED[1] requires a well-informed and determined patient who has family, friends, or others who can provide emotional and physical support; it’s also crucial that the patient have access to clinical caregivers who can provide palliative or hospice support.	1	2
Schwarz (2011) USA [22]	84-year-old woman with cancerous pelvic tumor metastasized throughout her abdomen; no further treatment following the initial surgery possible	She moved in a luxurious senior living facility and began to receive home hospice care (based on her daughter’s initiative)<br>VSED[1] would be the solution for her, as she had reached the conclusion that the burdens of living outweighed any benefit. But the hospice team denied supporting her, as they might lose their jobs. Her daughter denied as well being involved, as she sees a great legal risk for herself.<br>After the medical hospice doctor assured them of the legality of the patients’ choice, the hospice team and her daughter returned to support her in the dying process.<br>She died peacefully on her 8th day of her fast.	VSED[1] is a process that unfolds over time and requires thoughtful planning and support if the patient is to achieve a successful outcome – which is peaceful death that occurs within days to three weeks after beginning the fast.	1	2

[1]VSED = Voluntary stopping of eating and drinking

Reviews

Authors, nationality	Objective	Search / Sample	Data collection / Measurement	Analysis	Results	Conclusion	Evaluation Method. Quality	Relevance
Bernat et al. (1993) USA [16]	Suggesting that educating chronically and terminally ill patients about the feasibility of PRHN[1] can empower them to control their own destiny	Not stated	Not stated	Narrative description	PRHN[1] is an example of voluntary passive euthanasia ("letting die").<br>There is consensus of experienced health care staff that terminally ill patients dying of dehydration or lack of nutrition do not suffer if treated adequately.<br>PRHN[1] does not require the permission of a physician or legal approval. Physicians have a moral and legal obligation to respect the patients' refusal.<br>Physicians have an important responsibility to provide adequate symptom control for patients dying of PRHN[1].	There is no reason why patients who choose PRHN[1] should not have the same rights as the terminally ill who refuse life-sustaining therapies.	1	2
Byock (1995) USA [21]	Not stated	Not stated	Not stated	Narrative description	General impression among hospice workers "starvation and dehydration do not contribute to suffering " vs. general impression among the public "starvation and dehydration are terrible ways to die"	"Thirst" most often refers to a sensation of dryness in the mouth and throat rather than an experienced need to ingest a volume of fluid.<br>The decision to refuse food and fluids comes more from a felt sense of "being done".	1	2
Jansen (2004) New York, USA [18]	Discussing some of the ethical complications in view of the practice of VSED[2]	Not stated	Not stated	Narrative description	Significant ethical difficulties arise, when physicians, whose moral beliefs are against VSED[2], find themselves in situations where they must decide to take an active role in the decision-making process of a patient engaging VSED[2].	There is no moral safe harbour in care at the end-of-life, including VSED[2].	1	2
Quill et al. (1997) USA [20]	Comparing VSED[2], TS[3], PAS[4], and VAE[5] as potential interventions of last resort for competent, terminally ill patients	Not stated	Not stated	Narrative description	Refusing of life-prolonging interventions by a competent, informed patient is widely established; VSED[2] is seen as an extension of that right; no participation of a physician is needed.<br>But VSED[2] can last for weeks and in the beginning increase suffering if the patient experiences thirst or hunger.	VSED[2] is probably legal and widely accepted by hospice and palliative care workers. But some have still moral objections or legal fears concerning VSED[2].	1	2
Quill et al. (2000) USA [42]	Defining TS[3] and VRFF[6], distinguish them from standard palliative care and PAS[4], illustrate real clinical scenarios, provide potential clinical	Not stated	Not stated	Narrative description	In the case of VRFF[6],<br>-Palliative care must be available, and unable to adequately relieve current suffering<br>-Symptoms are unacceptable for the patient<br>-The terminal prognosis has been set for a few weeks to months	When unacceptable suffering persists despite best palliative efforts, VRFF[6] is imperfect but a useful last-resort option.	1	2

	guidelines, and explore their moral and legal status				-The patient should be competent and fully informed<br>-Patients' family and other health care provider are involved in the decision-making process<br>-Second opinion should be obtain from other experts			
Rady et al. (2012) Netherland [43]	Comment on: (1) unbearable suffering as an indication for PAD[7]; (2) VRFF[6] as a means to hasten death; (3) efficacy of CDS[8] for optimal control of distress from VRFF[6]; (4) bundling assisted dying with palliative and hospice care	Not stated	Not stated	Narrative description	VRFF is legal, but can result in a slow dying process.<br>The use of sedatives with VRFF is commensurate with euthanasia, because the clinical efficacy of CDS remains unproven.<br>Terminating life or assisted dying should not be equated with palliative or hospice care.	Because empirical evidence is absent in view of CDS[8], VRFF[6] with sedation can be considered as a cruel and inhumane method of terminating life.	1	1
Schwarz (2007) USA [41]	Exploring the moral and legal status of VSED[2]	Not stated	Not stated	Narrative description	VSED[2] has the advantage of relying solely on a competent patient's decision to control his/her end-of-life process.	Health care professionals are not obligated to participate in VSED[2], but they should facilitate the transfer of the patient's care to other health care members.	1	2
Yale Ohio, USA (2005) [45]	Not stated	Not stated	Not stated	Narrative description	VRFF[6] seems to be the most humane and ethical path to the alleviation of intractable suffering, but VRFF[6] in palliative care is understudied.	There is a need for clinical standards of practice for patients who choose VRFF[6].	1	2

[1]PRHN = Patients refusal of hydration and nutrition; [2]VSED = Voluntary stopping of eating and drinking; [3]TS = Terminal sedation; [4]PAS = Physician-assisted suicide; [5]VAE = Voluntary active euthanasia; [6]VRFF = Voluntary refusal of food and fluids; [7]PAD = Physician-assisted dying; [8]CDS = Continuous deep sedation
